# Deubiquitinase USP10 promotes osteosarcoma autophagy and progression through regulating GSK3β-ULK1 axis

**DOI:** 10.1186/s13578-024-01291-9

**Published:** 2024-09-02

**Authors:** Zuxi Feng, Yanghuan Ou, Xueqiang Deng, Minghao Deng, Xiaohua Yan, Leifeng Chen, Fan Zhou, Liang Hao

**Affiliations:** 1 Departments of Orthopedics, the 2st Affiliated Hospital, Jiangxi Medical College, Nanchang University, Nanchang, 330000 Jiangxi Province China; 2 Nottingham Trent University, Clifton, Nottingham, NG11 8NS UK; 3 Department of Biochemistry and Molecular Biology, School of Basic Medical Sciences, Nanchang University Jiangxi Medical College, Nanchang, 330031 China; 4 Department of Oncology, The Second Affiliated Hospital of Nanchang University, Nanchang, 330006 China; 5 Medical Center for Cardiovascular Diseases, Neurological Diseases and Tumors of Jiangxi Province, The Second Affiliated Hospital of Nanchang University, Nanchang, 330006 China; 6 Laboratory of Translational Medicine, National Cancer Center/National Clinical Research Center for Cancer/Cancer Hospital, Chinese Academy of Medical Sciences and Peking Union Medical College, Beijing, 100021 China; 7 Department of General Surgery, The Second Affiliated Hospital of Nanchang University, 1 Minde Road, Nanchang, 330006 Jiangxi People’s Republic of China

**Keywords:** Osteosarcoma, USP10, ULK1, GSK3β

## Abstract

**Background:**

Deubiquitinating enzymes (DUBs) are pivotal in maintaining cell homeostasis by regulating substrate protein ubiquitination in both healthy and cancer cells. Ubiquitin-specific protease 10 (USP10) belongs to the DUB family. In this study, we investigated the clinical and pathological significance of USP10 and Unc-51-like autophagy activating kinase 1 (ULK1) in osteosarcoma (OS), as well as the mechanism of USP10 action in ULK1-mediated autophagy and disease progression.

**Results:**

The analysis of OS and adjacent normal tissues demonstrated that USP10 and ULK1 were significantly overexpressed in OS, and a positive association between their expression and malignant properties was observed. USP10 knockdown in OS cells reduced ULK1 mRNA and protein expression, whereas USP10 overexpression increased ULK1 mRNA and protein expression. In vitro experiments showed that USP10 induced autophagy, cell proliferation, and invasion by enhancing ULK1 expression in OS cell lines. Furthermore, we found that the regulation of ULK1-mediated autophagy, cell proliferation, and invasion in OS by USP10 was dependent on glycogen synthase kinase 3β (GSK3β) activity. Mechanistically, USP10 promoted ULK1 transcription by interacting with and stabilising GSK3β through deubiquitination, which, in turn, increased the activity of the ULK1 promoter, thereby accelerating OS progression. Using a xenograft mouse model, we showed that Spautin-1, a small-molecule inhibitor targeting USP10, significantly reduced OS development, with its anti-tumour activity significantly enhanced when combined with the chemotherapeutic agent cisplatin.

**Conclusion:**

Collectively, we demonstrated that the USP10-GSK3β-ULK1 axis promoted autophagy, cell proliferation, and invasion in OS. The findings imply that targeting USP10 may offer a promising therapeutic avenue for treating OS.

**Supplementary Information:**

The online version contains supplementary material available at 10.1186/s13578-024-01291-9.

## Introduction

Osteosarcoma (OS), a common malignant bone tumor that affects young adults, is characterized by a high metastasis rates and poor prognosis [1]. OS has a tendency to metastasize, particularly to the lungs, becoming the leading cause of mortality in patients with OS [2]. Prior to the development of chemotherapy, surgery was the predominant treatment for OS, resulting in low survival rates [3]. Although chemotherapy combined with surgery is generally successful for treating non-metastatic OS, effective treatment options for patients with metastatic or recurrent OS remain a major clinical challenge [4].

Ubiquitin-specific protease 10 (USP10) is a member of the deubiquitinating enzyme family, characterized by the classic USP domain, which is responsible for deubiquitination [5]. USP10 deubiquitinases numerous tumor substrates, such as p53, KLF4, YAP/TAZ, and Raf-1, to regulate tumor growth and progression [6–9]. USP10 also affects cell fate and tumor progression via the regulation of autophagy, deubiquitinating LC3B to stabilize its protein levels [10]. The aforementioned studies indicate that USP10 exerts a significant influence on both autophagy and the progression of cancer. Nevertheless, the precise mechanism of USP10 in OS remains ambiguous.

The cytoplasmic kinase Unc-51-like autophagy-activating kinase 1 (ULK1) is essential for the initiation of autophagy, as it associates with the mammalian ATG1 protein complex [11, 12]. The N-terminal serine-threonine protein kinase domain of ULK1 is responsible for its kinase activity and substrate interaction, while the C-terminal interaction domain serves as a scaffold for the kinase domain [13]. Previous studies have shown that ULK1 directly binds to ATG8 and is degraded in lysosomes [14–16]. ULK1 protein levels can also be regulated by transcription [17] and phosphorylation: phosphorylation at Ser556 activates ULK1 to promote autophagy, demonstrating a tumor-suppressive effect in gastric cancer [18]. During glucose starvation, AMPK phosphorylates ULK1 at Ser317 and Ser777, directly activating autophagy. In the presence of glucose, mTOR phosphorylates ULK1 at Ser757, preventing ULK1 and AMPK interaction and thereby suppressing ULK1 activation [19]. Here, we demonstrated that USP10 knockdown inhibited ULK1 expression in OS cells. Therefore, we hypothesized that USP10 could affect OS autophagy and progression by regulating ULK1 expression.

In this study, we identified a novel role of USP10 in OS and demonstrated that the USP10-GSK3β-ULK1 axis plays a critical role in triggering autophagy and progression in OS. Furthermore, targeting the USP10-GSK3β-ULK1 axis with the USP10 inhibitor Spautin-1 shows synergistic effects in combination with cisplatin therapy, potentially offering therapeutic advantages to patients with OS. In summary, our findings suggest that USP10 is a promising therapeutic target for the efficient management of OS.

## Materials and methods

### Cell lines and culture

Human OS cell lines, including U2OS, 143B, and MG-63, as well as the normal cell line hFOB 1.19, were obtained from the Shanghai Institute of Cell Biology (China). The HOS cell line was purchased from Procell Life Science and Technology Co. HEK293T cells were obtained from the American Type Culture Collection (ATCC, Manassas, VA, USA). U2OS, 143B, and HEK293T cells were cultured in Dulbecco’s Modified Eagle’s Medium (DMEM, ATCC) containing 10% fetal bovine serum (FBS) at 37 °C and 5% CO_2_, while HOS and MG-63 cells were cultured in Eagle’s minimum essential medium (MEM) (Procell, Wuhan, China) containing 10% FBS at 37 °C and 5% CO_2_. All experiments were conducted using mycoplasma-free cells. Authentication procedures were conducted for all cell lines within the preceding 3 years. Short tandem repeat (STR) analysis was used to authenticate all acquired cell lines. The detailed authentication process was as follows: an appropriate number of cells (1 × 10^6^) was used for DNA extraction using Chelex100, and twenty STR loci and gender identification loci were amplified using the CELLID System. PCR product detection was conducted utilizing an ABI 3130xl genetic analyzer. The detection outcomes were then analyzed using Gene Mapper IDX (Applied Biosystems) and compared with data from ATCC, DSMZ, JCRB, ExPASy, and other relevant databases.

### Human tissue specimens

Patients undergoing surgery at the Second Affiliated Hospital and the First Affiliated Hospital of Nanchang University generously contributed human OS specimens, including adjacent tissues. Each specimen was subjected to a thorough pathological examination to establish a definitive diagnosis. The trial complied with the principles delineated in the Declaration of Helsinki and received approval from the Medical Ethics Committee of Nanchang University. Moreover, all patients provided informed consent prior to participation. Subsequently, the samples were preserved at −80 °C.

### Plasmids and antibodies

Flag-USP10-U1 (1-798aa), Flag-USP10-U2 (1-100aa), Flag-USP10-U3 (101-414aa), Flag-USP10-U4 (415-798aa), HA-GSK3β-G1 (1-420aa), HA-GSK3β-G2 (1-55aa), HA-GSK3β-G3 (56-340aa), HA-GSK3β-G4 (341-420aa), and plasmids for ULK1 overexpression, were acquired from Gene Chem and Gene-Pharma (Shanghai, China), respectively.

The following antibodies were used: Anti-USP10 (1:2000, ab109219, Abcam); anti-GSK3β (1:1000, ab32391, Abcam); anti-GSK3β (1:5000, #12456, CST); anti-ULK1 (1:1000, #8054, CST); anti-phospho-ULK1 (Ser555) (1:1000, #5869, T); anti-ULK1 (1:1000, sc-390904, Santa Cruz); anti-Flag (1:1000, #8146, CST); anti-HA (1;1000, #3724); anti-ubiquitin-linkage specific K63 (1:1000, ab179434, Abcam); anti-ubiquitin-linkage specific K48 (1:1000, ab140601, Abcam); anti-LC3B (1:1000, #3868, CST); anti-GAPDH (1:5000, ab8245, Abcam); anti-β-tubulin (1:5000, abs830032, Absin); anti-p62 (1:5000, ab109012, Abcam); anti-ubiquitin (1:1000, 10201-2-AP, Proteintech).

### Lentivirus infection

RFP-GFP-LC3 and short hairpin RNA (shRNA) lentivirus for USP10, GSK3β, as well as a negative control were purchased from Gene-Pharma (Shanghai, China). Lipofectamine 3000 (Invitrogen) was used to transduce OS cells for 48 h using serial dilutions of the lentiviral supernatant. Cells were selected using 3 μg/mL puromycin for 4 weeks, during which transfection efficiency was assessed through western blotting and qRT-PCR.

### Immunoprecipitation and pulldown assays

The cells underwent lysis using RIPA lysis buffer (Solarbio) supplemented with PMSF (Solarbio). Following centrifugation of the cell lysate at 10,000 × g for 10 min, the supernatant was harvested and subsequently incubated overnight at 4 °C with primary antibodies and protein A/G agarose beads (Santa Cruz). Immunocomplexes were subjected to three rounds of washing with 500 µL PBS, followed by centrifugation at 3000 rpm for 3 min each. Protein levels in both the lysates and immunocomplexes were evaluated via western blotting using appropriate primary antibodies.

For pull-down assays, in vitro purified Flag-USP10 (Sigma-Aldrich) was incubated with purified HA-GSK3β (Sigma-Aldrich) from HEK293T cells at 4 °C with rotation overnight. Beads underwent four rinses with RIPA buffer before undergoing western blotting analysis.

### Protein half-life assay

143B and U2OS cells, both with and without USP10 overexpression or silencing, were treated with cycloheximide (CHX) (Sigma, 10 mg/mL) for the specified time periods; collected protein samples were evaluated for GSK3β half-life.

### In vivo ubiquitination and deubiquitination assays

For the in vivo deubiquitination assessment, 143B and U2OS cells were exposed to 20 µM proteasome inhibitor MG132 (Selleck.cn) for 6 h, lysed with RIPA buffer containing protease inhibitors, subjected to immunoprecipitation using specific antibodies, followed by western blotting. Cells with or without USP10 knockdown, transfected with Flag-USP10 combined with additional constructs or Spautin-1, were used in these experiments.

### Transmission electron microscopy

OS cell lines stably expressing the specified constructs were cultured for 48 h in 10 cm plates. The cells were harvested and subsequently suspended in a 2% glutaraldehyde solution before being fixed overnight at 4 °C. Following fixation, ultrathin slices were prepared using an ultramicrotome (Leica EM UC7). The slices underwent staining with 2% uranyl acetate and lead citrate before being examined under an electron microscope (HITACHI HT7700).

### Colony formation assay

OS cells were seeded at a low density of 600–1200 cells per well in six-well plates and cultured for approximately 2 weeks. Following the incubation period, the cell colonies were washed with PBS washing, fixed in 4% paraformaldehyde for 20 min, stained with crystal violet, and then documented and enumerated.

### Transwell assay

OS cells underwent trypsinization, were resuspended in serum-free medium, and subsequent counted. The upper chamber received 200 µL of medium containing 10% FBS, while the bottom chamber was filled with 600 μL of the cell suspension. After a 36-h incubation period, the cells were fixed with 4% paraformaldehyde for 20 min and stained with 0.1% crystal violet for 30 min. The chambers were then rinsed three times with PBS before microscopic examination and cell counting.

### Real-time RT-PCR

Total RNA was obtained employing the Trizol reagent (Invitrogen). Reverse transcription was performed using the Prime Script TMRT kit (Takara, RR047A) and the Taq II Kit, and PCR amplification was conducted using SYBR Premix Ex according to the manufacturer’s instructions. Human primers (both forward and reverse) utilized were as follows:

USP10 (CCUUUGAGCCCACAUAUAUTT and AUAUAUGUGGGCUCAAAGGTT).

GSK3β (GTTAGCAGAGACAAGGACGGCA and GCAATACTTTCTTGATGGCGAC).

ULK1 (AAGTTCGAGTTCTCCCGCAA and ATAACCAGGTAGACAGAATTAGCCA).

GAPDH (GGAAGCTTGTCATCAATGGAAATC and TGATGACCCTTTTGGCTCCC).

LC3B (ACCCTGAGTCTTCTCTTCAGG and AGTTTACAGTCAGGGCCGTT).

### Immunohistochemistry (IHC) and haematoxylin & eosin (H&E) staining

Paired samples of OS and corresponding adjacent normal tissues were fixed in 10% formalin, followed by paraffin embedding, sectioning, and de-paraffinization. IHC was conducted utilizing anti-USP10 antibody (1:200, ab109219; Abcam) and anti-ULK1 antibody (1:100, sc-390904; Santa Cruz Biotechnology).

For Hematoxylin and Eosin (H&E) staining, the tissues underwent fixation in 10% formalin, dehydration, paraffin embedding, and subsequent sectioning (5–8 μm). The H&E staining was carried out following a standard protocol, and images were captured using a microscope.

### Tumour xenograft

The animal experiments were approved by the Nanchang University Committee and the Department of Animal Care and Use. Male BALB/c-nu/nu mice, aged 6–8 weeks, were used. Each group of mice (n = 6 per group) was injected with the indicated cell lines via tail vein injection. Mice were additionally administered cisplatin (25 mg/kg per day) and Spautin-1 (20 mg/kg per day) as indicated. Tumour volume and total body weight were measured every four days. After 35 days, tumours were excised from the dorsal areas of the mice, and their weights and volumes were recorded. Lung tissues dissected from mice injected with OS cells were examined for metastases.

### Statistical analysis

The analysis of results was conducted utilizing GraphPad Prism 6 software. Data are expressed as mean ± standard deviation (SD). Group disparities were evaluated through either one-way or two-way analysis of variance (ANOVA) or a two-sample t-test, with statistical significance set at *P* < 0.05.

## Results

### USP10 and ULK1 are highly expressed in human OS tissues

**Fig. 1:**
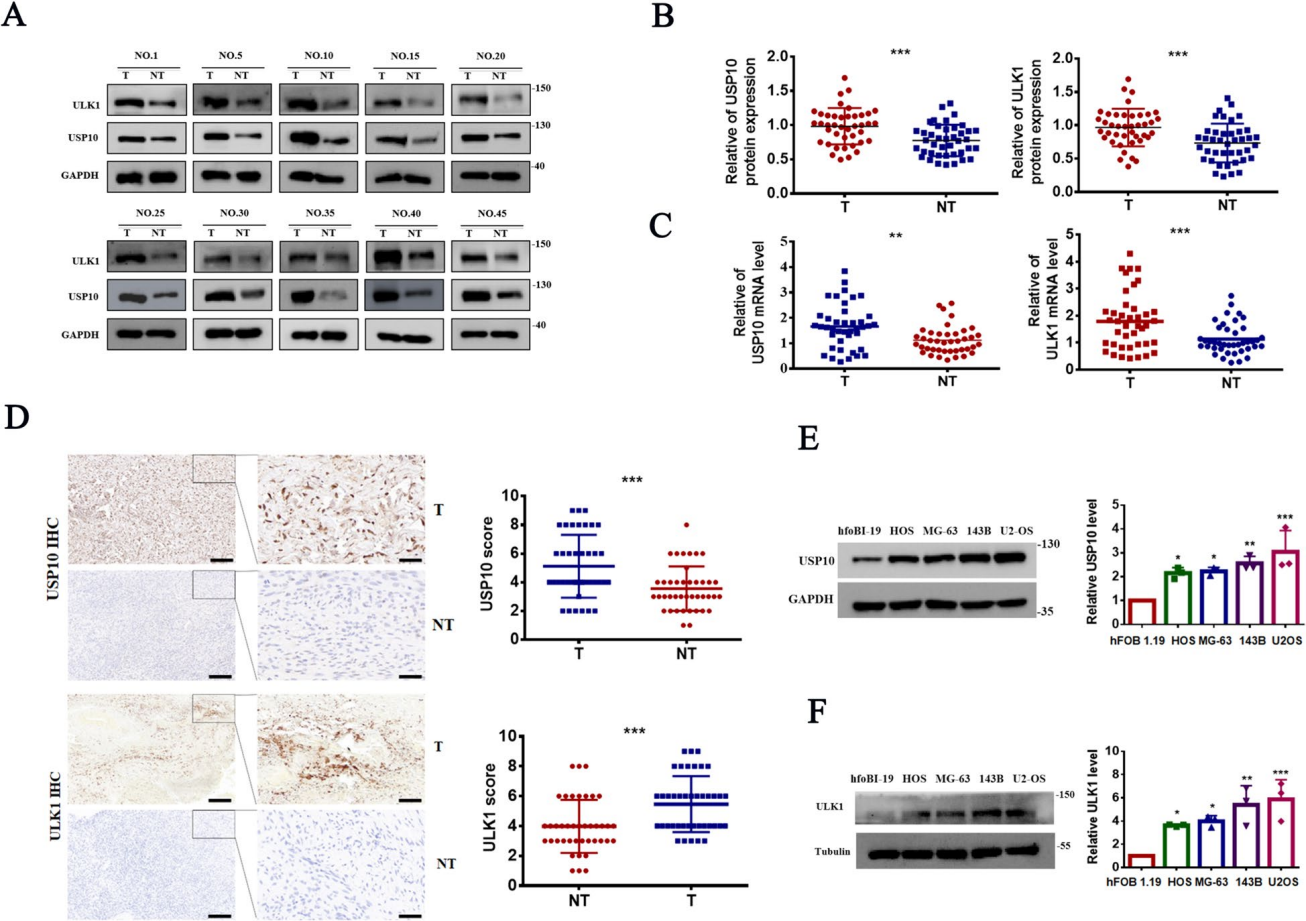
USP10 and ULK1 are highly expressed in human OS tissues. **A**–**B** Western blotting detected USP10 and ULK1 protein expression levels in 45 OS tissues and their paired non-cancerous counterparts. Consequently, the proteins quantification is displayed on the right (NT = paired non-tumor tissues, T = OS tissues). **C** q-RT-PCR analysis detected USP10 and ULK1 mRNA levels in 45 OS tissues and their corresponding non-tumor tissues. **D** Representative USP10 and ULK1 immunohistochemical staining images in OS (n = 45) and adjacent normal tissues (n = 41). An unpaired t-test was conducted to compare tumor tissues with adjacent normal tissues. Scale bars represent 50 μm. **E**–**F** q-RT-PCR and western blotting were performed to assess USP10 and ULK1 mRNA and protein levels in OS cell lines and normal hfoBI-19 osteoblasts

First, we assessed the expression levels of USP10 and ULK1 in OS (n = 45) and adjacent tissues. Our results demonstrated a substantial increase in both protein and mRNA expression levels of USP10 and ULK1 in OS tissues compared to adjacent tissues (Fig. 1A–C). IHC confirmed significantly higher expression levels of USP10 and ULK1 in OS tissues compared to adjacent tissue (Fig. 1D). Additionally, USP10 and ULK1 protein and mRNA expression levels were higher in OS cell lines compared to normal cells (Fig. 1E, F). These findings confirm elevated USP10 and ULK1 expression levels in human OS tissues.

### USP10 regulates autophagy, proliferation, and invasion in OS cells

**Fig. 2:**
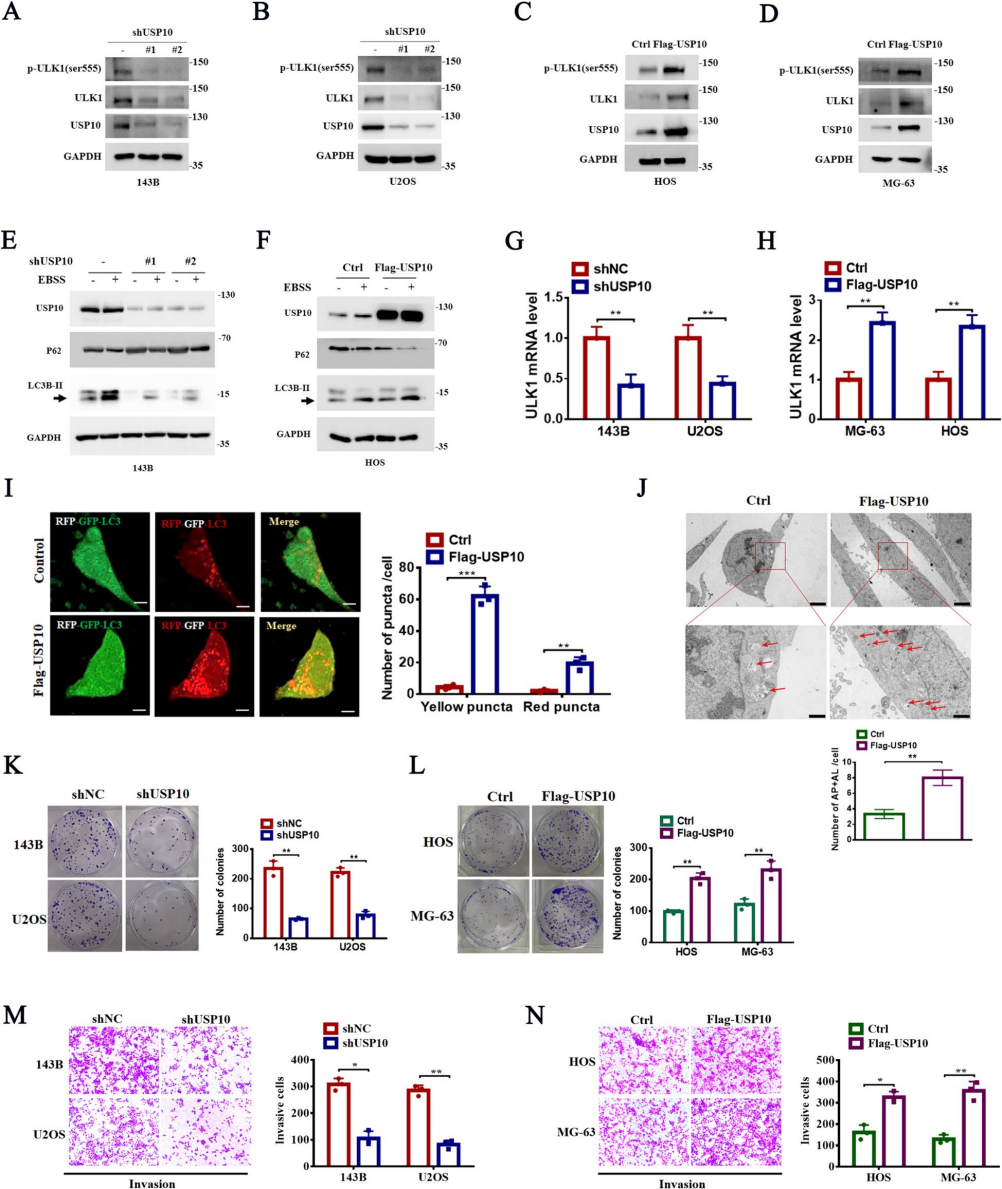
Stable knockdown of USP10 represses ULK1 expression and inhibits OS autophagy, proliferation, and invasion. **A**–**B** USP10 was downregulated using two independent shRNAs in 143B and U2OS cells. The protein levels of ULK1 and p-ULK1(ser555) were assessed through western blotting. **C**–**D** After overexpressing USP10 in MG-63 and HOS cells, the protein levels of ULK1 and p-ULK1(ser555) were assessed using western blotting. **E** USP10 knockdown in 143B cells by two independent shRNAs, which were then cultured in DMEM or EBSS for 6 h; LC3B-II and p62 protein concentrations by western blotting. **F** LC3B-II and p62 were analyzed in 143B cells stably expressing Flag-USP10 and cultured in DMEM or EBSS for 6 h. **G**–**H** The mRNA levels of ULK1 were examined using q-RT-PCR in 143B, U2OS, MG-63, and HOS cells with or without USP10 knockdown or overexpression. **I** Immunofluorescence analysis of autophagosome maturation was performed on RFP-GFP-LC3 cells stably overexpressing USP10 or in control groups cultured in DMEM for 4 h. Scale bars:10 μm, quantifying RFP-LC3-only puncta and RFP-GFP overlay puncta in indicated cells on the right. **J** HOS cells stably overexpressing USP10 or control were analyzed for autophagosomes and autolysosomes using TEM. The paper presents illustrative photographs and statistical analyses pertaining to autophagosomes and autolysosomes. Red arrows exhibit autophagosomes or autolysosomes. **K**–**L** USP10 overexpression in 143B cells and U2OS cells, cell growth was examined by colony formation. **M**–**N** The invasion of USP10 stable knockdown 143B cells were detected using a transwell assay

To investigate the role of USP10 in autophagy, proliferation, and invasion in OS, we silenced USP10 in 143B and U2OS cells and overexpressed it in MG-63 and HOS cells (Fig. S1A-B). Silencing USP10 significantly reduced the expression levels of ULK1 and p-ULK1 (Ser555) in OS cells (Fig. 2A, B and Fig. S1C), while its overexpression increased these levels (Fig. 2C, D and Fig. S1D-E). However, knockdown of ULK1 did not affect USP10 expression (Fig. S1F). Furthermore, silencing USP10 decreased LC3B-II levels and enhanced p62 accumulatio (Fig. 2E and Fig. S1C), whereas its overexpression increased LC3B-II and decreased p62 levels (Fig. 2F and Fig. S1D-E). qRT-PCR results showed significant reduction in ULK1 mRNA levels in USP10 knockdown cells and elevation in USP10 overexpressing cells (Fig. 2G, H). Additionally, we found that the expression level of the autophagy-related protein ATG13 is not regulated by USP10 (Fig. S1G-H). To further explore USP10's role in autophagosome maturation in OS, cells were transfected with tandem-tagged LC3 (RFP-GFP-LC3). USP10 overexpression promoted the maturation of autophagosomes into autolysosomes (Fig. 2I), while USP10 knockdown resulted in a decrease in this process (Fig. S1I). TEM analysis confirmed an increased number of autophagosomes in cells overexpressing USP10 (Fig. 2J). Colony formation assays showed that USP10 knockdown significantly decreased OS cell proliferation, whereas USP10 overexpression significantly increased proliferation (Fig. 2K, L and Fig. S1J-M). Finally, transwell assays demonstrated that USP10 knockdown significantly decreased cellular invasion, whereas ULK1 overexpression increased invasion (Fig. 2M, N and Fig. S1N-Q). These results underscore the critical role of USP10 in regulating autophagy, proliferation, and invasion in OS cells.

### ULK1 is essential for USP10-mediated autophagy, proliferation, and invasion in vitro and in vivo

**Fig. 3:**
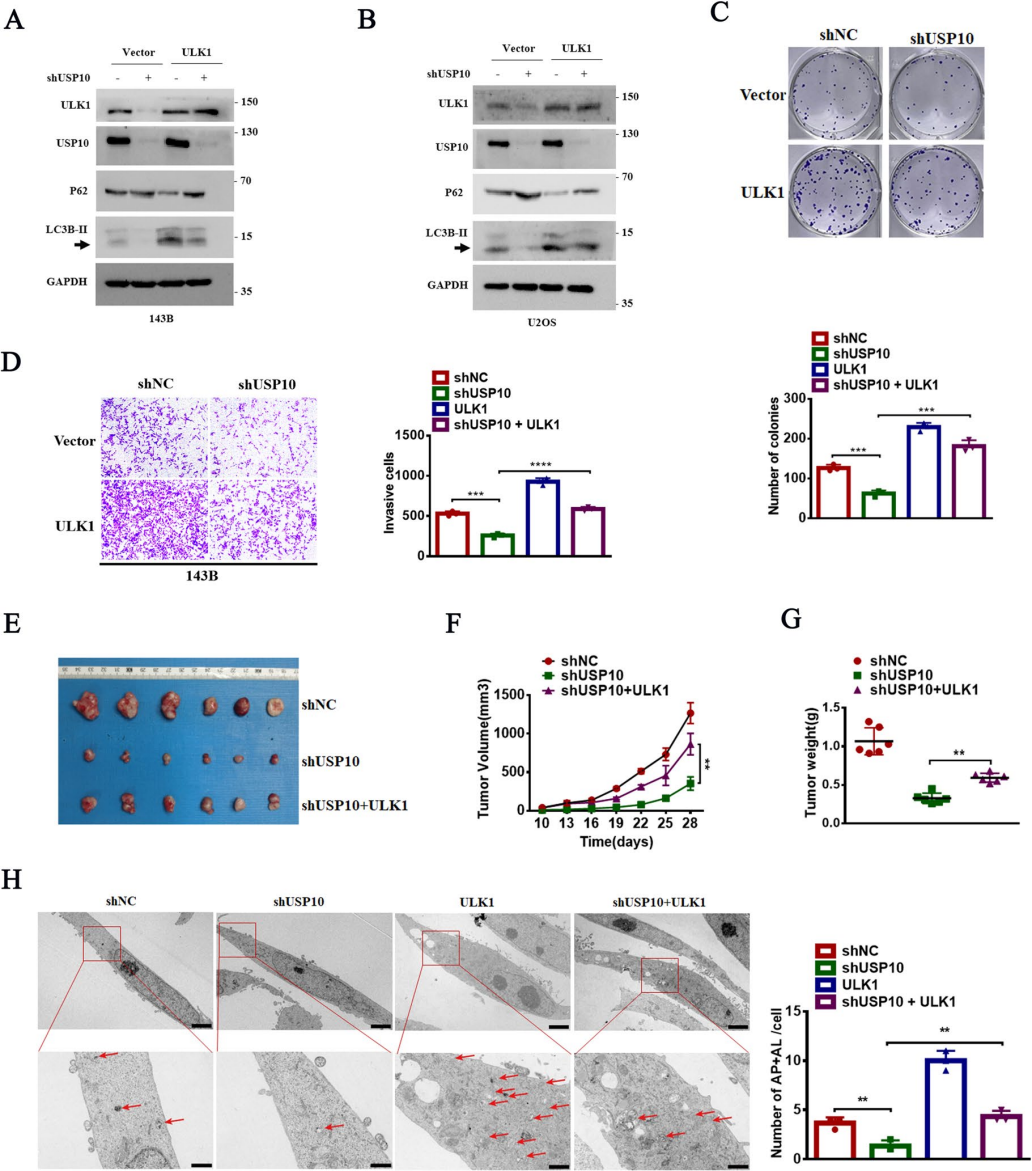
ULK1 is essential for USP10-mediated OS autophagy, proliferation, and invasion both in vitro and in vivo. **A**–**B** ULK1 was overexpressed in 143B cells with and without USP10 knockdown. The levels of ULK1, LC3B-II, and p62 were assessed through western blot analysis. **C**–**D** ULK1 was overexpressed in 143B cells, regardless of USP10 knockdown. Subsequently, we evaluated cell growth through colony formation assays (**C**), invasion was detected using a transwell assay (**D**). **E**–**G** The indicated osteosarcoma cells (2.0 × 10^6^ cells per mouse) were subcutaneously injected into nude mice (n = 6). Upon completion of the experiment, the tumors were surgically dissected, photographed (**E**), and weighed (**G**) Tumor volume was measured on the indicated days (**F**). **H** 143B cells stably expressed either shNC or shUSP10 with or without ULK1 overexpression were examined with autophagosomes and autolysosomes employing TEM. The figures presented include representative images and statistical analyses of autophagosomes and autolysosomes, with red arrows denoting autophagosomes. Scale bar: 20 μm

To investigate the impact of USP10 on OS development and autophagy through modulation of ULK1 expression, ULK1 was overexpressed in cells where USP10 was knocked down. Overexpression of ULK1 reversed the decrease in LC3B-II levels and the increase in p62 levels induced by USP10 knockdown (Fig. 3A, B). Additionally, ULK1 overexpression reversed the observed decrease in proliferation and invasion in USP10 knockdown cells (Fig. 3C, D). In vivo, tumor growth was inhibited following USP10 knockdown (Fig. 3E), with lower tumor weights and volumes were observed in the shUSP10 group compared to the shNC or shUSP10 + ULK1 groups (Fig. 3F, G). Furthermore, TEM results indicated that ULK1 overexpression reversed the decrease in autophagosome numbers induced by USP10 knockdown (Fig. 3H). Collectively, these findings suggest that USP10 promotes autophagy, proliferation, and invasion by upregulating ULK1 expression in OS, both in vivo and in vitro.

### USP10 regulates ULK1 expression in OS cells through GSK3β

**Fig. 4:**
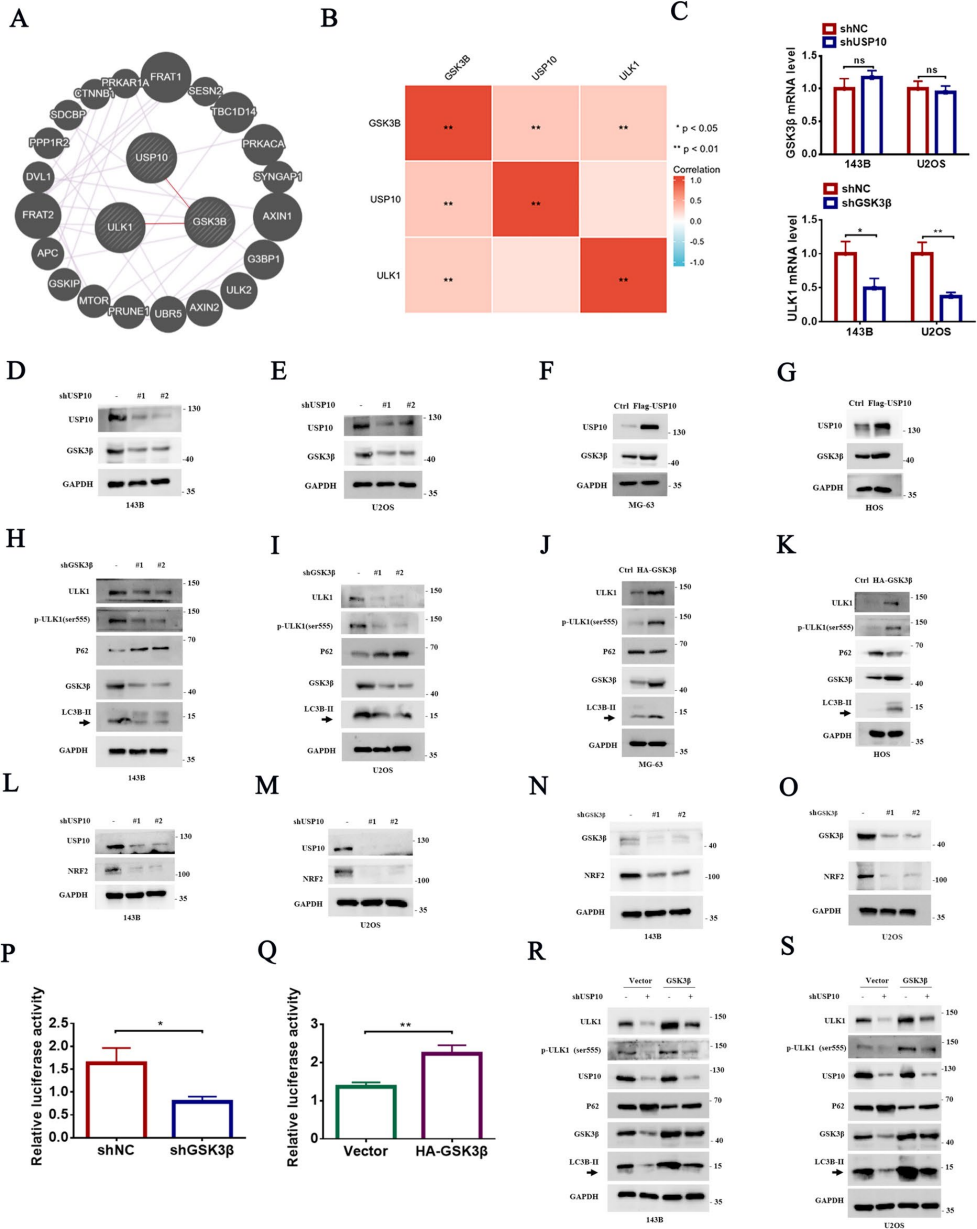
USP10 regulates ULK1 expression through GSK3β in OS cells. **A** Investigating the co-expression of USP10 and ULK1 genes using the Gene MANIA (http://genemania.org/) website. **B** A correlation analysis of three genes (USP10-GSK3β-ULK1) was conducted. The dataset of OS samples was obtained from the Gene Expression Omnibus (GEO) under accession number GSE42352. **C** The mRNA levels of GSK3β were examined in OS cells with or without USP10 knockdown, while the mRNA levels of ULK1 were assessed in OS cells with or without GSK3β knockdown. **D**–**E** USP10 expression was suppressed using two distinct shRNAs in 143B and U2OS cells, after which the protein levels of GSK3β were assessed. **F**–**G** The protein levels of GSK3β were assessed by western blotting after USP10 overexpression in MG-63 and HOS cells. **H**–**K** The protein levels of ULK1, p-ULK1(ser555), LC3B-II and p62 were detected by western blotting in OS cells with either GSK3β knockdown or GSK3β overexpression. **L**–**O** The protein levels of NRF2 were assessedby western blotting in OS cells with either USP10/GSK3β knockdown or overexpression. **P**–**Q** The promoter of ULK1 was transfected into OS cells with or without GSK3β knockdown or overexpression, and luciferase activity was subsequently measured. **R**–**S** Western blot analysis assessed the protein levels of ULK1, p-ULK1(Ser555), LC3B-II, and p62 in 143B and U2OS cells overexpressing GSK3β, with and without USP10 knockdown

To identify genes coexpressed with USP10 and ULK1, we utilized the GeneMANIA database (http://genemania.org/) **(**Fig. 4A**)**. We also conducted a correlation analysis on USP10, GSK3β, and ULK1 using Gene Expression Omnibus (GEO) datasets associated with OS. Our results demonstrated that GSK3β expression in OS was positively associated with USP10 and ULK1 expression (Fig. 4B). Silencing USP10 did not affect GSK3β mRNA levels, whereas GSK3β knockdown decreased ULK1 mRNA expression (Fig. 4C). Furthermore, USP10 knockdown decreased GSK3β expression in OS cells (Fig. 4D, E and Fig. S2A), whereas USP10 overexpression significantly upregulated GSK3β (Fig. 4F, G and Fig. S2B-C). To validate these findings, GSK3β was knocked down in 143B and U2OS cells and overexpressed GSK3β in MG-63 and HOS cells (Fig. S2D-E). Furthermore, GSK3β knockdown significantly reduced ULK1 and p-ULK1 (Ser555) levels (Fig. 4H, I and Fig. S2F), whereas GSK3β overexpression increased ULK1 and p-ULK1 levels (Ser555) (Fig. 4J, K and Fig. S2G-H). However, the overexpression of ULK1 did not affect the expression of GSK3β (Fig. S2I-J). We hypothesized that GSK3β affects ULK1 transcription by regulating downstream transcription factors, such as nuclear factor erythroid-derived 2-like 2 (NRF2) [20]. As expected, USP10 or GSK3β knockdown significantly reduced NRF2 expression (Fig. 4L–O), whereas USP10 or GSK3β overexpression increased NRF2 expression (Fig. S2K-N). Furthermore, knocking down NRF2 in OS cell lines resulted in decreased expression of ULK1, while overexpressing NRF2 increased ULK1 expression (Fig. S2O-R). OS cells, with or without GSK3β knockdown or overexpression, were transfected with pGL3-based luciferase reporter plasmids containing the ULK1 promoter. ULK1 promoter luciferase activity was significantly reduced in GSK3β knockdown cells and increased in GSK3β overexpressing cells (Fig. 4P, Q). We then evaluated the effect of GSK3β overexpression on autophagy in USP10 knockdown 143B and U2OS cells. Our results indicated that GSK3β overexpression reversed the USP10 knockdown-induced decrease of LC3B-II and ULK1 levels, and the increase in p62 levels (Fig. 4R, S).

### ULK1-mediated USP10 function in OS is GSK3β-dependent

**Fig. 5:**
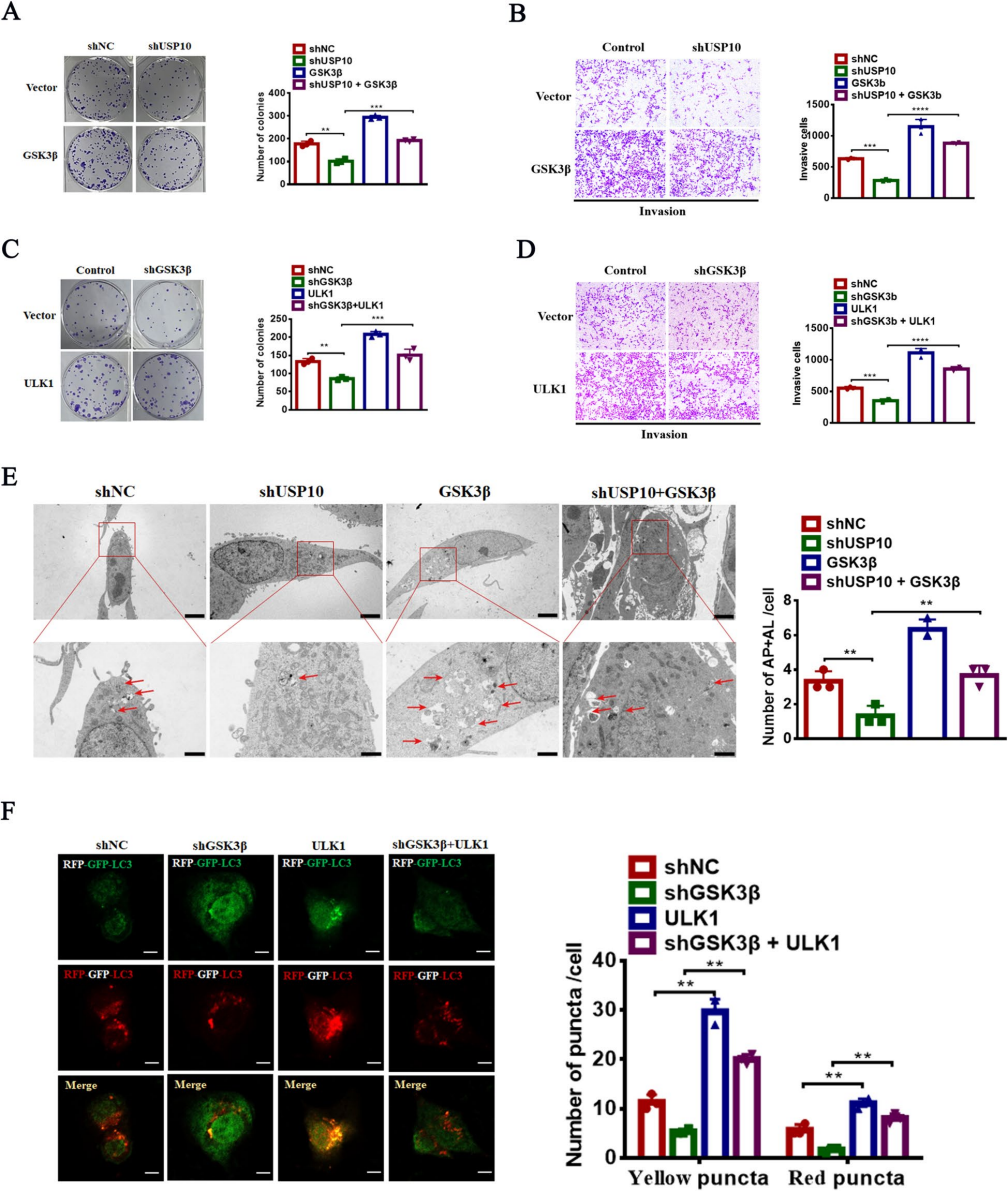
The regulation of ULK1-mediated malignant behaviors in OS by USP10 is dependent on GSK3β. **A**–**B** We evaluated cell proliferation and invasion in 143B cells with or without USP10 knockdown by assessing the impact of overexpressed GSK3β. This assessment was conducted through colony formation assays (**A**) and transwell assays (**B**). **C**–**D** We examined cell proliferation and invasion in 143B cells with overexpressed ULK1, both with and without USP10 knockdown, using colony formation (**C**) and transwell assays (**D**). **E** Both USP10-knockdown and non-knockdown 143B cells were subjected to GSK3β overexpression, and subsequently analyzed for the presence of autophagosomes and autolysosomes using transmission electron microscopy (TEM). The figures presented herein include representative images along with statistical analyses of autophagosomes and autolysosomes, with red arrows denoting the autophagosomes. The scale bar for all images is set at 20 μm. **F** Autophagosome maturation analyzed by immunofluorescence in RFP-GFP-LC3 cells stably expressing shGSK3β, with or without ULK1 overexpression and cultured in DMEM for 6 h. Scale bars: 10 μm. Quantification of RFP-LC3-only puncta and RFP-GFP overlay puncta in indicated cells

Functional experiments demonstrated that USP10 knockdown reduced proliferation and invasion, which was reversed by GSK3β overexpression (Fig. 5A, B). Next, we evaluated the effect of ULK1 overexpression on autophagy and tumor progression in 143B and U2OS cells with GSK3β knockdown. Our findings demonstrated that ULK1 overexpression reversed the effects of GSK3β knockdown on proliferation and invasion (Fig. 5C, D). TEM showed that GSK3β overexpression enhanced autophagy and rescued the decrease in autophagosome number induced by USP10 knockdown (Fig. 5E). Furthermore, the ectopic expression of ULK1 induced autophagy in GSK3β knockdown cells (Fig. 5F). Collectively, our results showed that USP10 stimulates OS autophagy and tumor progression by upregulating the GSK3β-ULK1 axis.

### USP10 interacts with GSK3β

**Fig. 6:**
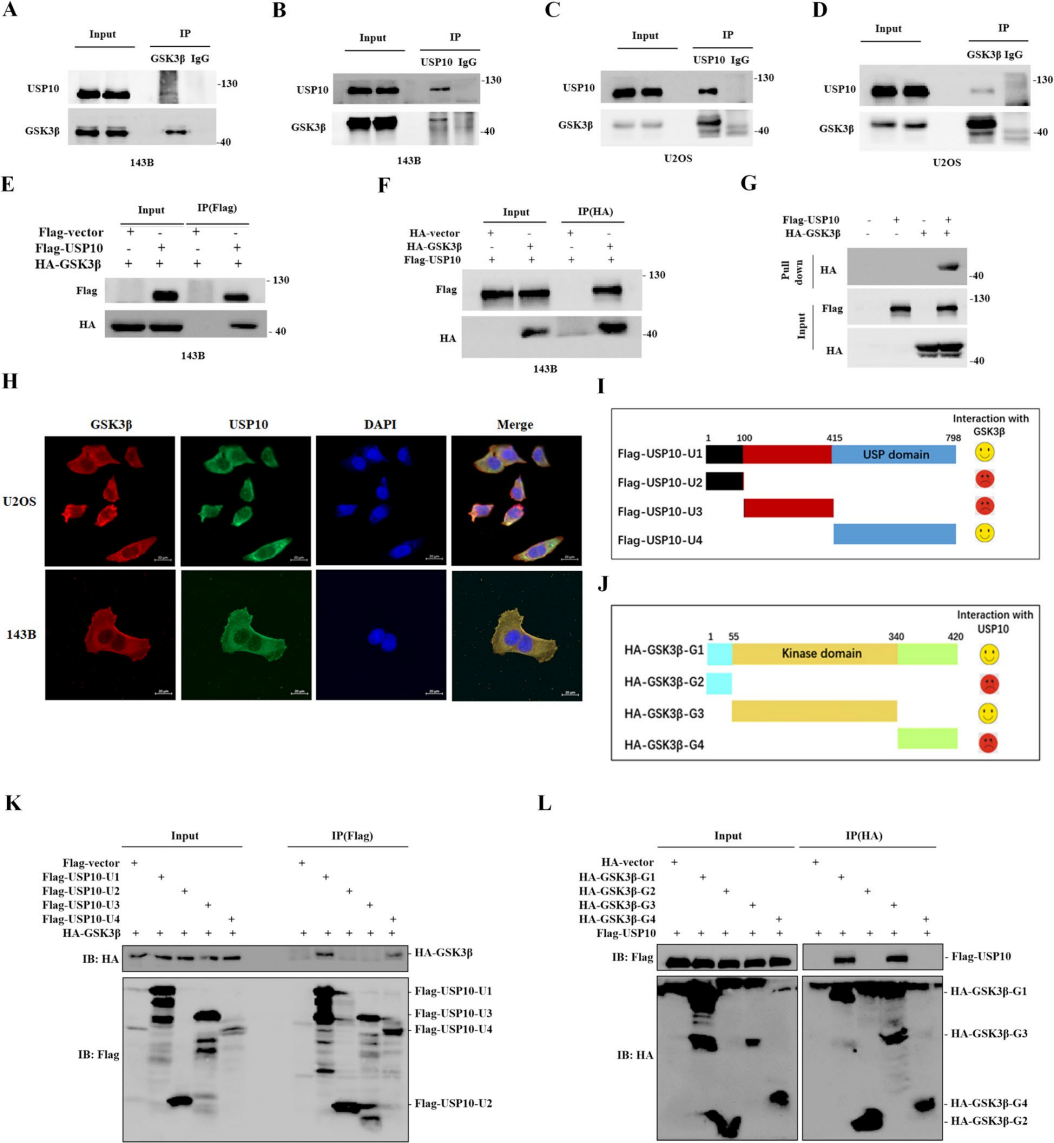
USP10 interacts with GSK3β. **A**–**D** Cell lysates from 143B and U2OS cells underwent immunoprecipitation (IP) using anti-IgG, anti-USP10 and anti-GSK3β antibodies. The immunoprecipitates were subsequently detected via western blotting employing the specified antibodies. **E**–**F** Reciprocal immunoprecipitation analysis was conducted to examine the interaction between USP10 and GSK3β in 143B cells transfected with FLAG-USP10 and HA-GSK3β. **G** The purified Flag-USP10 from HEK293T cells was subjected to an in vitro interaction assay with separately purified HA-GSK3β. Following incubation, the sepharose beads was probed using the anti-Flag antibody. **H** 143B and U2OS cells were fixed and stained with USP10 antibodies (green) and GSK3β antibodies (red), and nuclei were counterstained with DAPI (blue). The scale bar represents 20 μm. **I**–**J** This schematic representation illustrates the structural composition of USP10 and GSK3β. **K**–**L** Immunoprecipitation and western blot analyses were performed on cell lysates from HEK293T cells transfected with the specified constructs

We hypothesized that USP10 interacts with GSK3β to induce its deubiquitination. Indeed, endogenous GSK3β was coimmunoprecipitated with endogenous USP10 (Fig. 6A–D). Ectopically expressed Flag-USP10 was bound to HA-GSK3β (Fig. 6E, F), and an in vitro pull-down assay confirmed USP10 interacted with GSK3β (Fig. 6G). Immunofluorescence revealed colocalization of USP10 and GSK3β in 143B and U2OS cells (Fig. 6H). To investigate USP10 and GSK3β binding sites, HA-tagged GSK3β and USP10 domains (Fig. 6I), as well as FLAG-tagged USP10 and several GSK3β domains, were utilized in HEK293FT cells (Fig. 6J). Co-immunoprecipitation experiments showed that the USP10 USP domain interacts with GSK3β (Fig. 6K), and the GSK3β kinase domain (56-340aa) interacts with USP10 (Fig. 6L).

### USP10 maintains GSK3β stability via deubiquitination

**Fig. 7:**
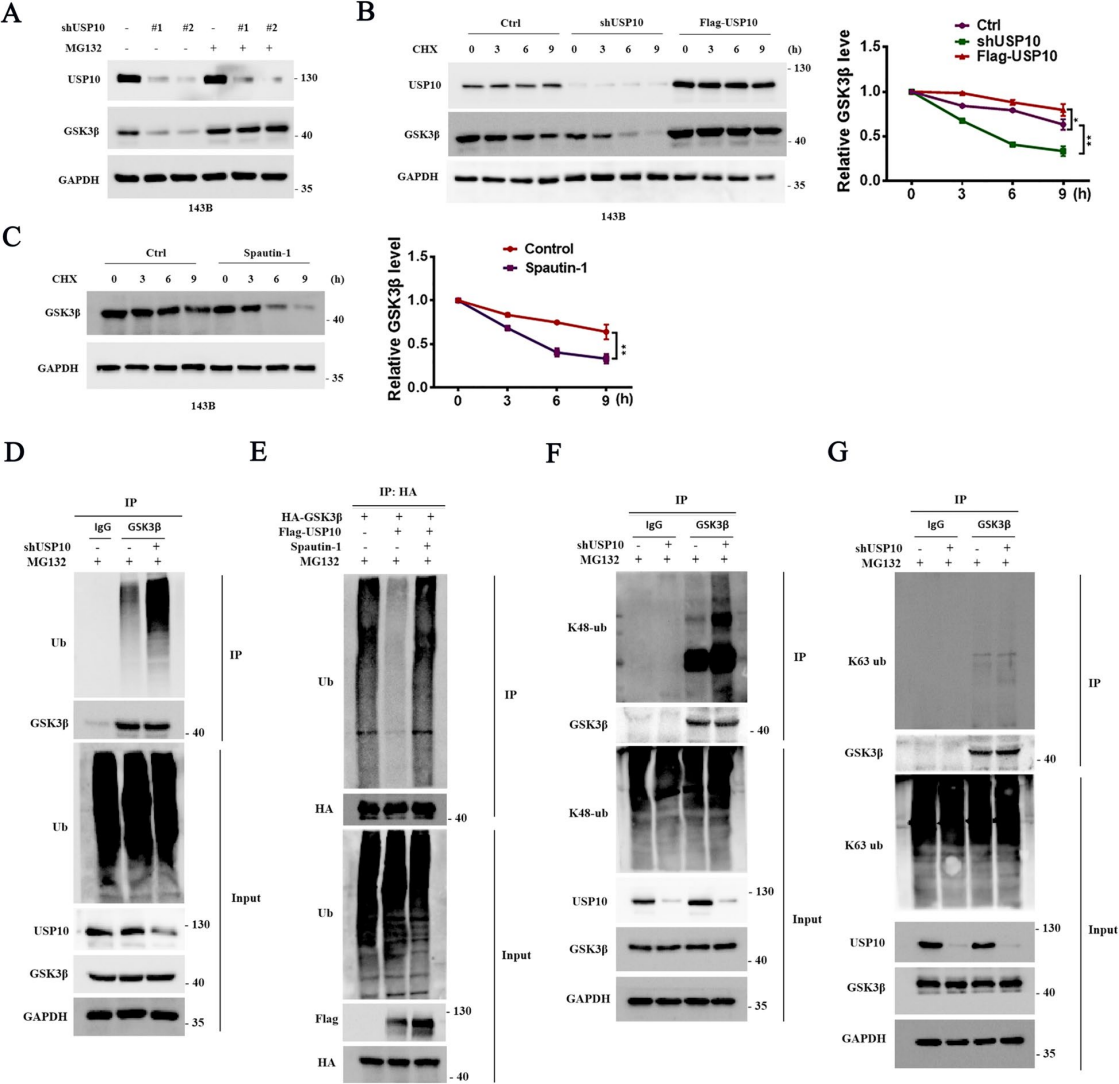
USP10 maintains GSK3β stability through deubiquitinating GSK3β. **A** The western blot analysis examined the expression of GSK3β in 143B cells treated with 15 µM MG132 for 6 h, with and without knockdown of USP10. **B** 143B cells treated with CHX (10 mg/mL), with or without USP10 overexpression or knockdown, were analyzed at the indicated times. The cell lysates were subjected to analysis with the specified antibodies to measure the half-life of GSK3β. **C** 143B cells were exposed to spautin-1 (10 µM) and cycloheximide (CHX) (10 mg/mL) for the specified durations. Subsequently, cell lysates were probed with designated antibodies to determine the decay rate of GSK3β. **D** MG132-treated 143B cells, with or without USP10 knockdown, were analyzed for 6 h. Whole-cell lysates underwent immunoprecipitation using either anti-GSK3β or anti-IgG antibodies. Subsequent western blot analysis utilized anti-Ub antibodies to detect ubiquitylated GSK3β. **E** 143B cells overexpressing USP10 or treated with spautin-1 were subjected to MG132 treatment and transfected with HA-GSK3β for 6 h before collection. Whole-cell lysates underwent immunoprecipitation with an HA antibody, followed by western blot analysis using an anti-Ub antibody to confirm the presence of ubiquitylated GSK3β. **F**–**G** 143B cells treated with MG132 for 6 h, with or without USP10 knockdown, underwent whole-cell lysate immunoprecipitation using an anti-GSK3β antibody. This was followed by western blot analysis employing either anti-Ubiquitin (specific to K48 linkage) or anti-Ubiquitin (specific to K63 linkage) antibodies to ascertain the nature of GSK3β ubiquitination

Numerous studies have established that GSK3β is degraded by the proteasome [21–24]. Therefore, OS cells with or without USP10 knockdown underwent exposure to the proteasomal inhibitor MG132. Our data showed that MG132 inhibited GSK3β downregulation induced by USP10 knockdown (Fig. 7A). Next, we exposed OS cells to the protein synthesis inhibitor CHX and showed that increased USP10 expression significantly improved the stability of endogenous GSK3β (Fig. 7B). Furthermore, the degradation rate of GSK3β was significantly increased in cells treated with the USP10 inhibitor Spautin-1 (Fig. 7C). Since USP10 is a deubiquitinase, we hypothesized that USP10 affects the ubiquitination of GSK3β. As expected, USP10 knockdown increased the levels of endogenous GSK3β polyubiquitination (Fig. 7D), while USP10 overexpression reduced them (Fig. 7E). USP10-overexpressing cells treated with Spautin-1 had higher polyubiquitination levels of ectopically expressed GSK3β (Fig. 7E). Furthermore, USP10 knockdown increased K48- but not K63-linked GSK3β ubiquitination (Fig. 7F, G). Furthermore, USP10 overexpression suppressed ubiquitination of GSK3β linked to K48 but not K63-linked GSK3β ubiquitination (Fig. S3A-B). Collectively, these results indicated that USP10 stabilised GSK3β via deubiquitination.

### USP10 inhibitor Spautin-1 inhibits OS growth in vitro and in vivo

**Fig. 8:**
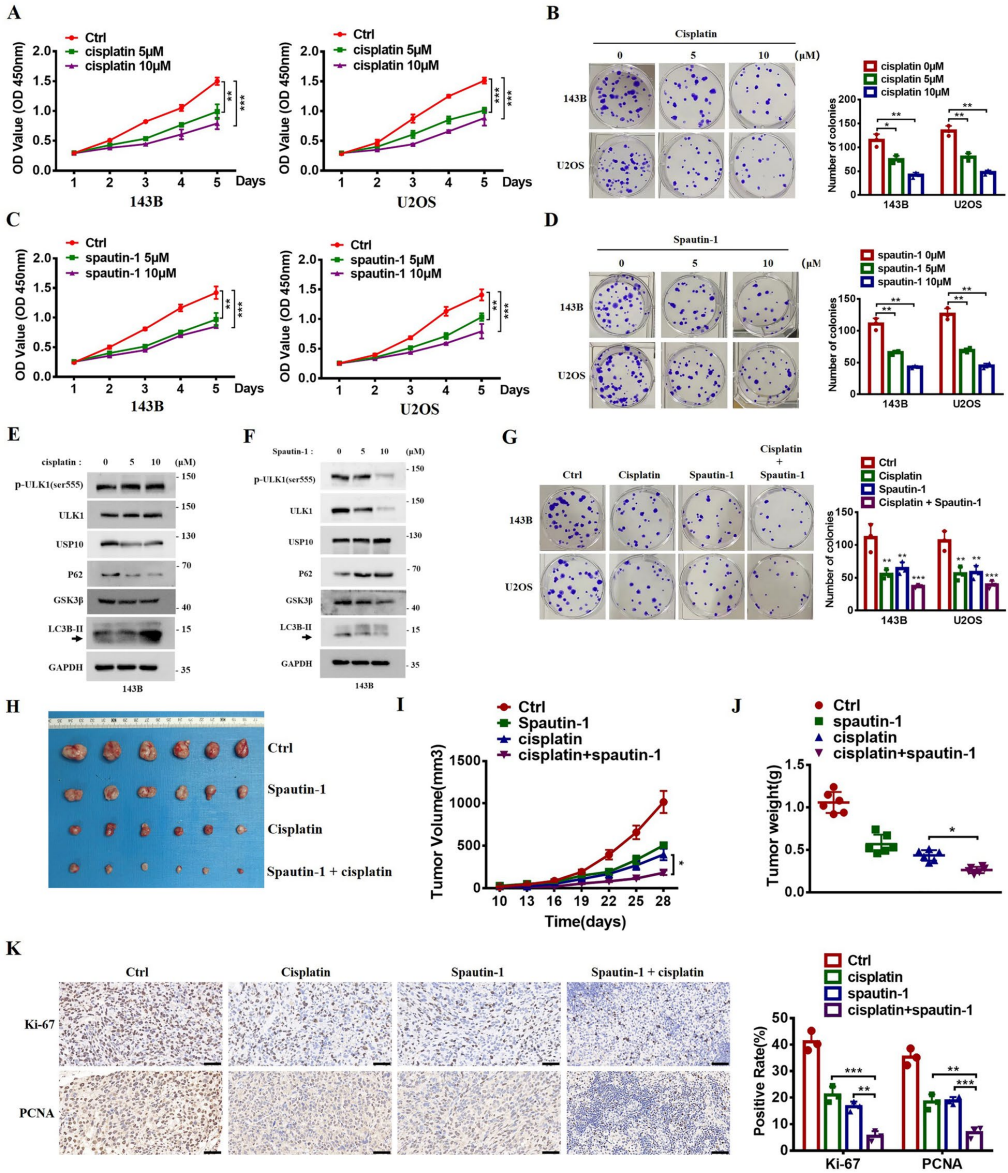
USP10 inhibitor Spautin-1 inhibits OS growth in vitro and in vivo. **A**–**B** The 143B and U2OS cell lines underwent treatment with cisplatin across specified concentrations (0, 5, and 10 μM) for durations spanning 0–3 days or 7–14 days. CCK8 assay (**A**) and colony formation assay (**B**) was performed according to the instructions. Colony numbers were quantified using Image J software. **C**–**D** 143B and U2OS cells underwent treatment with Spautin-1 at specified concentrations (0, 5, and 10 μM) for 0–3 days or 7–14 days. CCK8 assay (**C**) and colony formation assay (**D**) was performed according to the instructions. Colony numbers were quantified. **E** 143B cells underwent treatment with Spautin-1 at specified concentrations (0, 5, and 10 μM) for 48 h. The impact of Spautin-1 on the expression levels of ULK1, p-ULK1 (ser555), GSK3β, LC3B-II, and p62 were examined by western blotting. **F** 143B cells were subjected to treatment with cisplatin at specified concentrations (0, 5, and 10 μM) for a duration of 48 h. Subsequently, western blotting was employed to assess the protein levels of USP10, ULK1, p-ULK1 (ser555), GSK3β, LC3B-II, and p62. **G** The cell lines 143B and U2OS were subjected to treatments involving different concentrations of cisplatin (0 μM, 5 μM, and 10 μM), Spautin-1 (0 μM, 5 μM, and 10 μM), or a combined treatment of cisplatin and spautin-1 over a duration of 7 to 14 days. Colonies were stained and fixed. Colony numbers were quantified as shown on the right. **H**–**J** Nude mice received injections with 143B cells (2 × 10^6^ cells/0.2 mL). They were subsequently randomized to receive either intraperitoneal injections of Spautin-1 (20 mg/kg daily), gavage administration of cisplatin (25 mg/kg daily), or both, over a 15-day period. Tumor volumes (**J**) were measured at indicated time (10, 13, 16, 19, 22, 25, and 28 days). All tumors were excised, and their weights (**I**) were measured on the final day (mean values ± SEM, n = 6). Images of the tumors are shown in panel (**H**). **K** Immunohistochemistry was used to detect Ki67 (upper) and PCNA (lower), with representative images provided. Quantification of Ki67 and PCNA is shown on the right

Subsequently, we investigated the impact of Spautin-1 and cisplatin on the proliferation of OS cells. Both Spautin-1 and cisplatin demonstrated dose-dependent inhibition of proliferation in 143B and U2OS cells (Fig. 8A–D). Treatment with Spautin-1 decreased levels of LC3B-II, GSK3β, ULK1, and ULK1 (p-Ser555) while increasing p62 levels in a dose-dependent manner (Fig. 8E). In contrast, cisplatin increased LC3B-II expression while reducing levels of GSK3β, USP10, and p62; ULK1 and p-ULK1 (Ser555) levels remained unchanged (Fig. 8F). Unexpectedly, when GSK3β was knocked down, Spautin-1 did not further inhibit ULK1 and autophagy (Fig. S3C). Simultaneous treatment with Spautin-1 and cisplatin markedly enhanced inhibition of cell growth compared to other groups (Fig. 8G); this was consistent with results from the colony formation assay (Fig. 8G). In vivo, xenograft assays demonstrated that co-treatment with cisplatin and Spautin-1 significantly inhibited tumor growth more effectively than other treatments (Fig. 8H–J). IHC analysis revealed a decrease in the expression levels of the proliferation markers Ki-67 and PCNA in the tumor tissues following treatments with Spautin-1 and cisplatin. Notably, the combination treatment of cisplatin and Spautin-1 exhibited a more pronounced reduction in tumor proliferation compared to either treatment alone (Fig. 8K). These results suggest that co-administration of Spautin-1 and cisplatin markedly reduced OS growth both in vitro and in vivo.

## Discussion

USP10 plays a critical role in tumor cell proliferation, metastasis, apoptosis, metabolism, and cancer progression by deubiquitinating a diverse array of substrates [8, 9, 25–30]. While other studies have reported that USP10 promotes autophagy in certain cells [6, 10, 31]; its exact mechanism in OS development remains unclear. In this study, we evaluated the involvement of USP10 in autophagy, growth, and invasion in OS cells. Our findings reveal that USP10 induces autophagy in OS cells. Furthermore, USP10 knockdown reduced OS carcinogenesis in both in vivo and in vitro settings. To explore the specific mechanism by which USP10 regulates autophagy in OS cells, we examined potential USP10 substrates.

ULK1 serves as a pivotal kinase crucial for initiating autophagy [32]. Numerous studies have documented that the suppression of ULK1 kinase activity results in reduced autophagy [33–35]. ULK1 plays various roles in cancer progression, promoting the development of pancreatic [36], colon [37, 38], high-grade serous ovarian [39], nasopharyngeal [40], and breast cancer [41]. However, contradictory findings suggest that ULK1 may suppress cancer development and metastasis in breast [42, 43] and gastric cancers [18]. Wu et al. found that knockdown of the oncogene Aurora-B inhibited the mTOR/ULK1 signalling pathway, as well as OS cell migration and invasion [44]. Additionally, long noncoding RNA SNHG6 was found to be overexpressed in OS and to regulate the miR-26a-5p/ULK1 axis post-transcriptionally, thereby enhancing OS cell proliferation, invasiveness, and apoptosis [45].

Here, we demonstrated that increasing ULK1 expression through USP10 upregulation rendered OS cells more susceptible to autophagy and tumor development. USP10 knockdown significantly decreased ULK1 and p-ULK1 (Ser555) levels, while USP10 overexpression significantly increased ULK1 and p-ULK1 (Ser555) levels, suggesting that USP10 regulated ULK1 transcription. As expected, USP10 knockdown downregulated ULK1 mRNA levels, whereas USP10 overexpression upregulated ULK1 mRNA levels, indicating that ULK1 induced autophagy in OS cells, and ULK1 overexpression reversed autophagy inhibition caused by USP10 knockdown. Both in vitro and in vivo experiments illustrated that overexpression of ULK1 restored USP10 knockdown-induced inhibition of OS cell proliferation and invasion. This finding confirms that USP10 promotes autophagy, growth, and invasion of OS cells through upregulation of ULK1 transcription.

To investigate regulatory mechanisms, we hypothesized the presence of an intermediate protein mediating USP10 regulation through ULK1. Using the GeneMANIA (http://genemania.org) database, we identified GSK3β, a serine/threonine kinase involved in cell growth, DNA repair, the cell cycle, signalling, and metabolism. The analysis of GEO dataset (GSE42352) demonstrated a positive correlation between USP10 and GSK3β, as well as GSK3β and ULK1expression, in OS. GSK3β is involved in canonical signalling pathways and cancer progression as a tumor suppressor; however, it also functions as an oncogene to accelerate tumor growth in pancreatic and colorectal cancer [46] and hepatocellular carcinoma [47]. GSK3β exhibits a dual role in cancer, including OS [48]. Several GSK3β inhibitors have been identified for their ability to suppress OS cell proliferation [49–52]. For example, the active form of GSK3β (p-Tyr216) was overexpressed, whereas the inactive form GSK3β (p-Ser9) was downregulated in all OS cell lines compared to hFOB1.19 osteoblasts. Additionally, silencing of GSK-3β by siRNA inhibited OS growth and induced apoptosis [53].

Our results also showed that GSK3β knockdown significantly decreased OS growth and metastasis. Furthermore, USP10 knockdown significantly downregulated GSK3β protein levels and increased its ubiquitination, despite no change in GSK3β mRNA levels. Subsequently, we demonstrated that USP10 interacted with GSK3β and identified the binding domains. GSK3β overexpression restored the inhibition of autophagy, growth, and metastasis induced by USP10 knockdown. Conversely, GSK3β knockdown decreased autophagy, growth, and invasion in OS, with these effects reversible by ULK1 overexpression. We also showed that GSK3β knockdown significantly reduced ULK1 and p-ULK1 (Ser555) levels. To investigate this new GSK3β-mediated mechanism of ULK1 regulation in OS, we performed luciferase assays and demonstrated that GSK3β induced ULK1 transcription by increasing ULK1 promoter activity. Previous studies have shown several transcription factors involved in the regulation of ULK1 transcription [54]. The transcription factor NRF2 is notably overexpressed in OS and its upregulation correlates with unfavorable prognosis and chemoresistance among OS patients [55–58]. The findings of our study demonstrate that the suppression of either USP10 or GSK3β leads to a reduction in NRF2 expression. Therefore, we proposed that NRF2 was the transcription factor involved in GSK3β-mediated regulation of ULK1 transcription.

Spautin-1 has shown efficacy in suppressing liver cancer metastasis in both in vivo and in vitro settings [59]. It also reduces melanoma cell growth and enhances cisplatin's anti-tumor properties when combined [60]. Consistent with prior research, we showed that Spautin-1 effectively suppressed OS cell proliferation. When combined with cisplatin, it inhibited OS growth in vitro and in vivo more effectively than either compound alone. Treatment with Spautin-1 in OS cells significantly decreased GSK3β and ULK1 expression. Cisplatin treatment, meanwhile, inhibited USP10 and GSK3β expression. In our study, Spautin-1 inhibited the expression of GSK3β and ULK1, but the knockdown of GSK3β did not enhance the inhibitory effect of Spautin-1 on autophagy. Therefore, Spautin-1 might influence the autophagy and proliferation of OS cells through other signaling pathways regulated by USP10. Our findings collectively demonstrate the significant role of USP10 in OS by activating the GSK3β-ULK1 axis, which in turn promotes autophagy and carcinogenesis.

## Conclusion

**Fig. 9:**
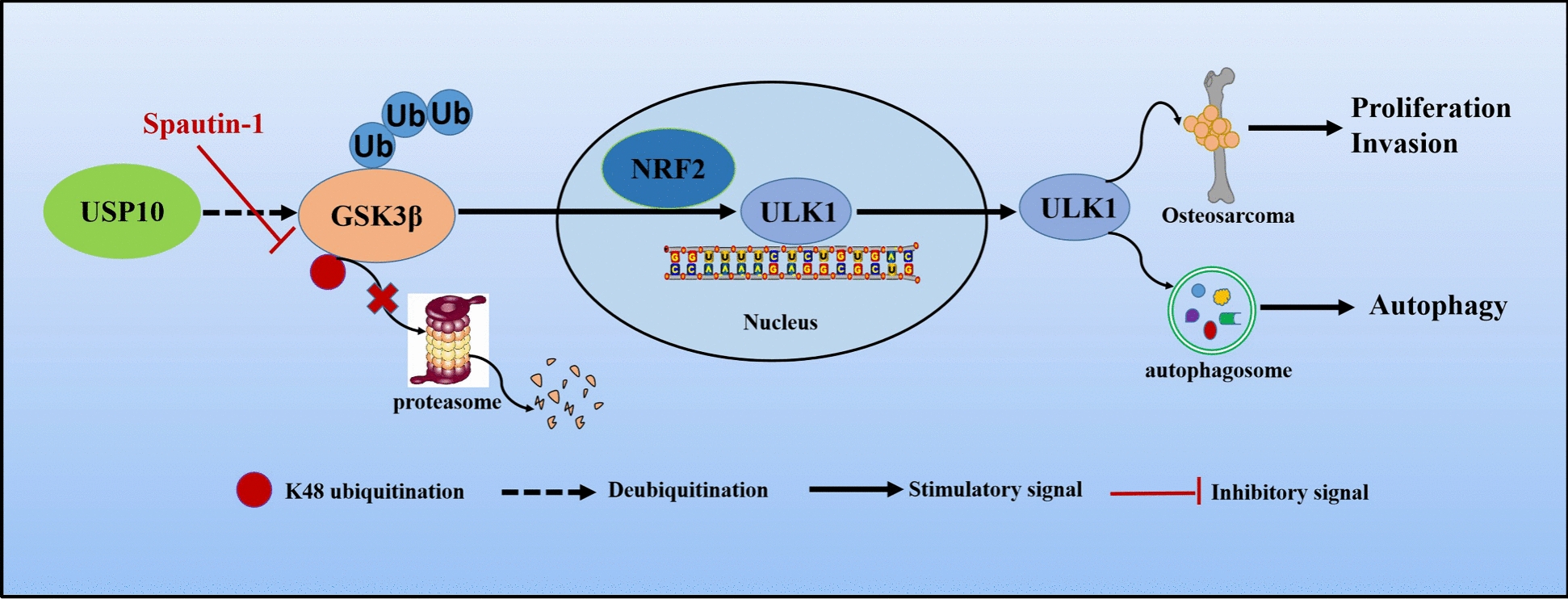
Schematic diagram illustrating the role of USP10 in promoting OS proliferation, invasion, and autophagy through GSK3β-ULK1 axis. A model has shown that USP10 interacts with GSK3β and stabilizes it, leading to the accumulation of GSK3β, which in turn transcriptionally upregulates ULK1 expression. Furthermore, functional experiments have demonstrated that the USP10-GSK3β-ULK1 axis promotes autophagy and tumor progression in OS. These phenomena can be suppressed by Spautin-1. Consequently, our proposed USP10-GSK3β-ULK1 axis offers new perspectives and evidence for molecularly targeted therapy in OS

In conclusion, we demonstrated that USP10 binds to GSK3β and stabilizes it through deubiquitination, inducing ULK1 transcription by enhancing its promoter activity. Furthermore, our experiments showed that the USP10-GSK3β-ULK1 axis was involved in autophagy and tumor progression in OS. Moreover, co-treatment of cisplatin with the USP10 inhibitor Spautin-1 significantly enhanced its anti-tumor effects, suggesting that targeting this axis could offer a new therapeutic approach for OS (Fig. 9).

### Supplementary Information

Supplementary material 1: Figure S1.The expression levels of USP10 in 143B and U2OS cells were evaluated by western blotting and q-RT-PCR following USP10 knockdown or overexpression.USP10 expression was downregulated using shRNAs in HOS and MG-63 cells. Subsequently, protein levels of LC3B-II, p62, ULK1, and p-ULK1were analyzed by western blotting.Upon USP10 overexpression in 143B and U2OS cells, the protein levels of LC3B-II, p62, ULK1, and p-ULK1were determined via western blotting.ULK1 was downregulated specifically in 143B cells. Subsequently, protein levels of USP10 were analyzed using western blotting.USP10 was downregulated or overexpressed specifically in 143B cells. Subsequently, protein levels of ATG13 were analyzed using western blotting.The process of autophagosome maturation was examined through immunofluorescence analysis in RFP-GFP-LC3 cells. These cells were stably transfected with either shUSP10 or shNC and subsequently cultured in EBSS for a duration of 4 h. Scale bars:10 μm. Quantifying RFP-LC3-only puncta and RFP-GFP overlay puncta in indicated cells.USP10 knockdown in HOS and MG-63 cells or USP10 overexpression in 143B cells and U2OS cells, cell growth was examined through colony formation.USP10 knockdown in HOS and MG-63 cells or USP10 overexpression in 143B cells and U2OS cells, cell invasion was detected using a transwell assay

Supplementary material 2: Figure S2.USP10 was downregulated in 143B cells. The protein levels of GSK3β were assessed through western blotting.After overexpressing USP10 in 143B and U2OS cells, the protein levels of GSK3β were assessed using western blotting. Western blotting and q-RT-PCR analysis of GSK3β expression in 143B and U2OS cells stably knocked down or overexpressed GSK3β.Western blotting analysis of ULK1 and p-ULK1expression in HOS and MG-63 cells stably knocked down or overexpressed GSK3β.Western blotting analysis of GSK3β expression in HOS and MG-63 cells stably overexpressed ULK1.Western blotting analysis of NRF2 expression in HOS and MG-63 cells stably overexpressed USP10.Western blotting analysis of NRF2 expression in HOS and MG-63 cells stably overexpressed GSK3β.Western blotting analysis of ULK1 expression in 143B and U2OS cells knockdown NRF2 by siRNA.Western blotting analysis of ULK1 expression in HOS and MG-63 cells stably overexpressed NRF2.

Supplementary material 3: Figure S3.143B cells, with or without USP10 overexpression, were treated with MG132 for 6 hours. Next, whole-cell lysates underwent immunoprecipitation with an anti-GSK3β antibody. Western blot analysis was performed using anti-Ubiquitin antibodies targeting either the K48 or K63 linkage to investigate GSK3β ubiquitination.Cells with stable knockdown of GSK3β were treated with spautin-1 at a concentration of 10 μM for 48 hours. Protein levels of ULK1, LC3B-II, and p62 were assessed using western blotting

## Data Availability

The data generated in this study are available upon request from the corresponding author.

## Abbreviations

USP10: Ubiquitin-specific protease 10
ULK1: Unc-51-like autophagy activating kinase 1
GSK3β: Glycogen synthase kinase 3β
OS: Osteosarcoma
DUBs: Deubiquitinating enzymes
AMPK: Adenosine 5 ‘-monophosphate (AMP)-activated protein kinase
mTOR: Mammalian target of rapamycin
NRF2: Nuclear Factor erythroid 2-Related Factor 2
LC3B: Microtubule-associated protein 1 light chain 3 beta
