# Supplementary figures and images for: Deubiquitinase USP10 promotes osteosarcoma autophagy and progression through regulating GSK3β-ULK1 axis
Source: Cell Biosci. 2024 Sep 2;14:111. doi: 10.1186/s13578-024-01291-9 (PMC11367994; doi:10.1186/s13578-024-01291-9)

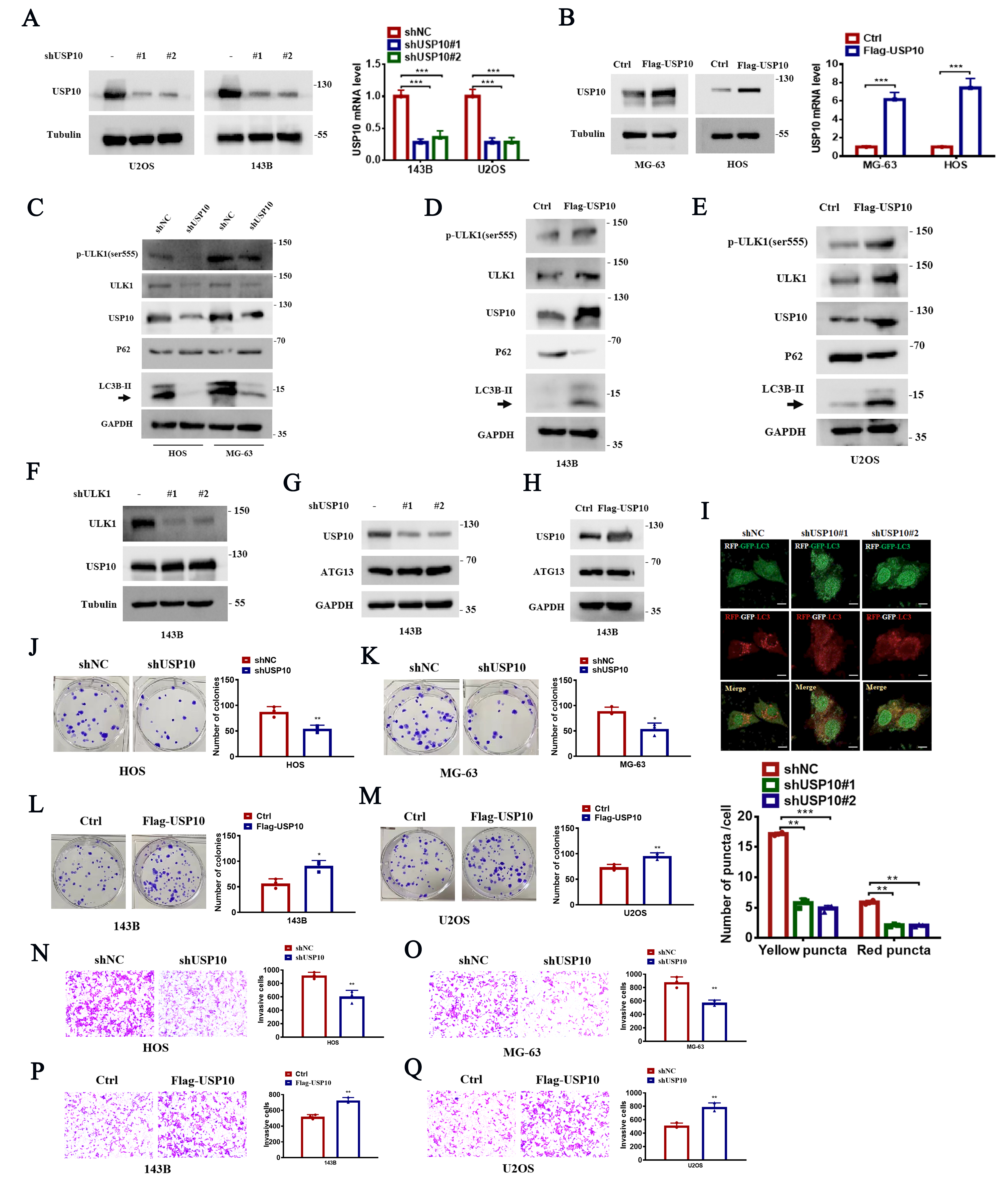

Supplement: Supplementary file 1 — Supplementary material 1: Figure S1.The expression levels of USP10 in 143B and U2OS cells were evaluated by western blotting and q-RT-PCR following USP10 knockdown or overexpression.USP10 expression was downregulated using shRNAs in HOS and MG-63 cells. Subsequently, protein levels of LC3B-II, p62, ULK1, and p-ULK1were analyzed by western blotting.Upon USP10 overexpression in 143B and U2OS cells, the protein levels of LC3B-II, p62, ULK1, and p-ULK1were determined via western blotting.ULK1 was downregulated specifically in 143B cells. Subsequently, protein levels of USP10 were analyzed using western blotting.USP10 was downregulated or overexpressed specifically in 143B cells. Subsequently, protein levels of ATG13 were analyzed using western blotting.The process of autophagosome maturation was examined through immunofluorescence analysis in RFP-GFP-LC3 cells. These cells were stably transfected with either shUSP10 or shNC and subsequently cultured in EBSS for a duration of 4 h. Scale bars:10 μm. Quantifying RFP-LC3-only puncta and RFP-GFP overlay puncta in indicated cells.USP10 knockdown in HOS and MG-63 cells or USP10 overexpression in 143B cells and U2OS cells, cell growth was examined through colony formation.USP10 knockdown in HOS and MG-63 cells or USP10 overexpression in 143B cells and U2OS cells, cell invasion was detected using a transwell assay [file 13578_2024_1291_MOESM1_ESM.tif]

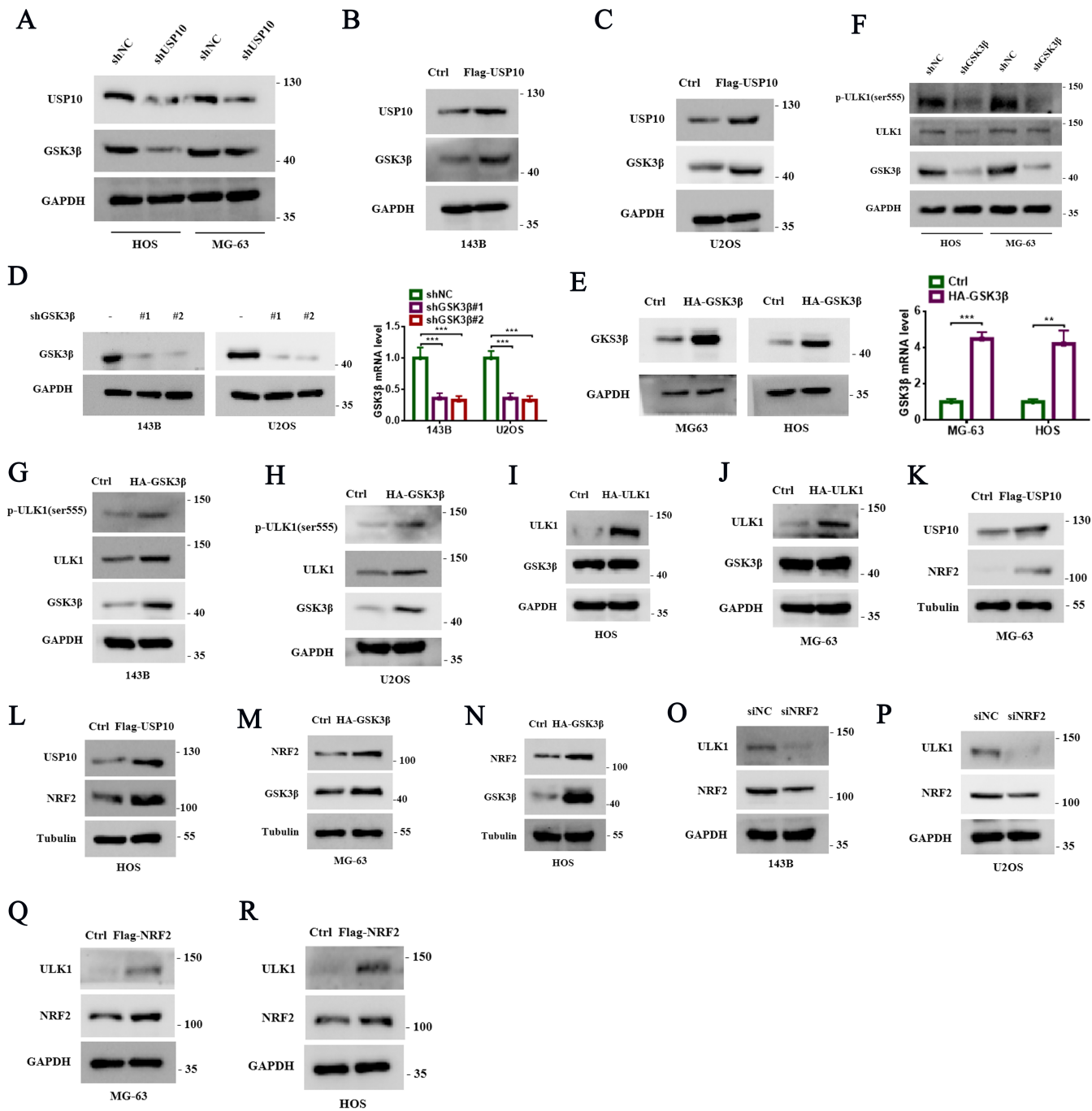

Supplement: Supplementary file 2 — Supplementary material 2: Figure S2.USP10 was downregulated in 143B cells. The protein levels of GSK3β were assessed through western blotting.After overexpressing USP10 in 143B and U2OS cells, the protein levels of GSK3β were assessed using western blotting. Western blotting and q-RT-PCR analysis of GSK3β expression in 143B and U2OS cells stably knocked down or overexpressed GSK3β.Western blotting analysis of ULK1 and p-ULK1expression in HOS and MG-63 cells stably knocked down or overexpressed GSK3β.Western blotting analysis of GSK3β expression in HOS and MG-63 cells stably overexpressed ULK1.Western blotting analysis of NRF2 expression in HOS and MG-63 cells stably overexpressed USP10.Western blotting analysis of NRF2 expression in HOS and MG-63 cells stably overexpressed GSK3β.Western blotting analysis of ULK1 expression in 143B and U2OS cells knockdown NRF2 by siRNA.Western blotting analysis of ULK1 expression in HOS and MG-63 cells stably overexpressed NRF2. [file 13578_2024_1291_MOESM2_ESM.pdf]

**A**

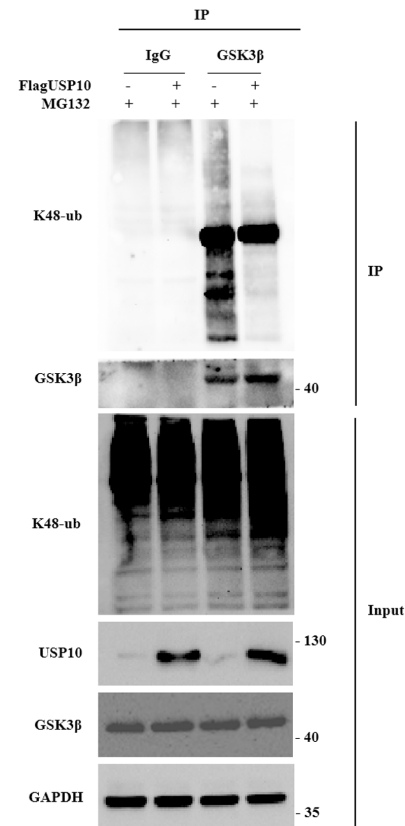

**B**

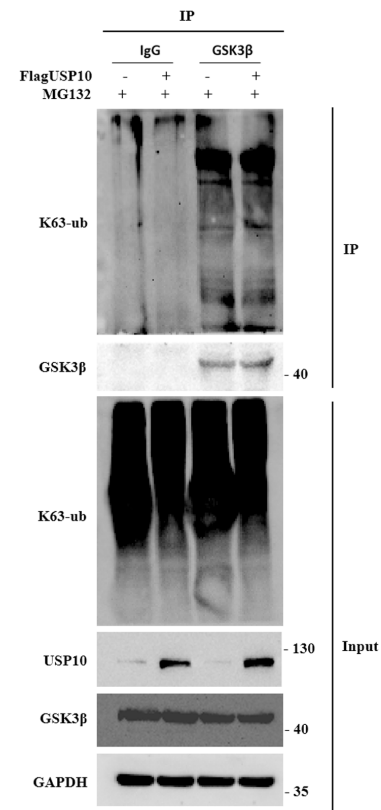

**C**

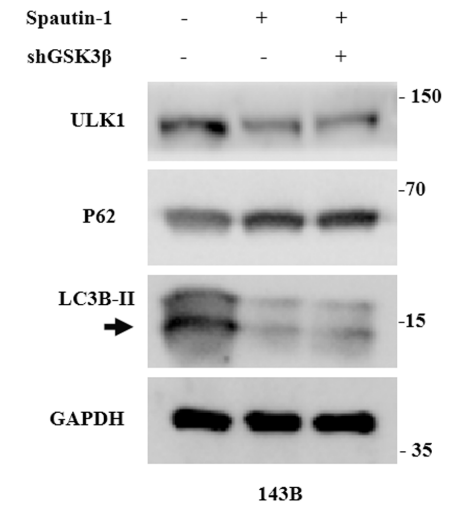

Supplement: Supplementary file 3 — Supplementary material 3: Figure S3.143B cells, with or without USP10 overexpression, were treated with MG132 for 6 hours. Next, whole-cell lysates underwent immunoprecipitation with an anti-GSK3β antibody. Western blot analysis was performed using anti-Ubiquitin antibodies targeting either the K48 or K63 linkage to investigate GSK3β ubiquitination.Cells with stable knockdown of GSK3β were treated with spautin-1 at a concentration of 10 μM for 48 hours. Protein levels of ULK1, LC3B-II, and p62 were assessed using western blotting [file 13578_2024_1291_MOESM3_ESM.pdf]
